# Multi Beam Dielectric Lens Antenna for 5G Base Station

**DOI:** 10.3390/s20205849

**Published:** 2020-10-16

**Authors:** Farizah Ansarudin, Tharek Abd Rahman, Yoshihide Yamada, Nurul Huda Abd Rahman, Kamilia Kamardin

**Affiliations:** 1 School of Electrical Engineering, Universiti Teknologi Malaysia, Johor 81310, Malaysia; tharek@utm.my; 2 Department of Electrical, Electronic & Systems Engineering, Faculty of Engineering & Built Environment, Universiti Kebangsaan Malaysia, Bangi, Selangor 43650, Malaysia; 3 Wireless Communication Centre, Universiti Teknologi Malaysia, Johor 81310, Malaysia; 4 Malaysia-Japan International Institute of Technology, Universiti Teknologi Malaysia, Kuala Lumpur 54100, Malaysia; yoshihide@utm.my (Y.Y.); kamilia@utm.my (K.K.); 5 Faculty of Electrical Engineering, Universiti Teknologi MARA, Shah Alam, Selangor 40450, Malaysia; nurulhuda0340@uitm.edu.my

**Keywords:** lens antenna, lens shaping method, straight-line condition, multi beam radiation pattern, wide angle range

## Abstract

In the 5G mobile system, new features such as millimetre wave operation, small cell size and multi beam are requested at base stations. At millimetre wave, the base station antennas become very small in size, which is about 30 cm; thus, dielectric lens antennas that have excellent multi beam radiation pattern performance are suitable candidates. For base station application, the lens antennas with small thickness and small curvature are requested for light weight and ease of installation. In this paper, a new lens shaping method for thin and small lens curvature is proposed. In order to develop the thin lens antenna, comparisons of antenna structures with conventional aperture distribution lens and Abbe’s sine lens are made. Moreover, multi beam radiation pattern of three types of lenses are compared. As a result, the thin and small curvature of the proposed lens and an excellent multi beam radiation pattern are ensured.

## 1. Introduction

Nowadays, the 5G mobile system is rapidly developing to achieve fast rate transmission, low latency, extremely high traffic volume density, super-dense connections and improved spectral energy, as well as cost efficiencies [1,2,3]. With the introduction of 5G, the mobile technology has new features such as millimetre wave operation, small cell size and multi beam base station antenna to meet massive multiple-input multiple-output (MIMO) requirements [4,5,6]. At millimetre wave, the base station antenna size is expected to be less than 30 cm, and due to the massive MIMO usage in 5G technology, the antenna system shall have excellent multi beam radiation patterns. Aperture antennas such as a dielectric lens and reflector can be among the alternatives proposed to replace the present array antenna system. Based on recent studies [7,8], the dielectric lens antenna is known to produce excellent multi beam patterns as compared to the reflector antenna. A Luneburg lens that is composed of spherical layered material has achieved a good multi beam radiation pattern in very wide-angle region [9]. However, having a Luneburg lens with continuously varying material permittivity as a function of the lens radius is quite difficult to accomplish in practice. As a result, many methods for these non-uniform geometries have been studied to simplify the lens geometry for various communication applications as well as its feeding network [10,11,12]. However, in those works, the capabilities of performing wide angular scanning for multi beam applications were not discussed in detail.

As for the case of a homogenous lens, the shaped lens, designed based on Abbe’s sine condition, has achieved good wide-angle multi beam characteristics [13]. In achieving excellent radiation patterns, lens surfaces were shaped by ray tracing based on the Geometrical Optics (GO) method [14,15,16,17], where, typically, lens shaping can be done by the aperture distribution method or Abbe’s sine method [18,19]. Shaped dielectric lens antennas were developed for the low side lobe radiation pattern and multi beam radiation pattern [20,21,22]. For low side lobe and multi beam shaping, different lens forming equations were employed. However, to satisfy the installation requirement, the previous lens shaping methods were not suitable. Furthermore, it was used for single beam radiation pattern synthesis only, and the applications were limited to airborne radar and vehicle’s collision avoidance system. In [23], multi beam radiation characteristics are studied sufficiently with Abbe’s sine case. However, for a practical base station usage, this lens shape was not suitable due to the large diameter of the circular lens and because it was operating at low frequency. In addition to that, the scanning angle was limited to 30° of angular range. Therefore, small lens thickness and small curvature are requested in a 5G lens antenna system to meet the requirements of multi beam scanning, ease-of-installation on a thin pole and lightweight structure. Furthermore, the 5G antenna system shall incorporate a massive multiple-input multiple-output (MIMO) feature; thus, an antenna with a wide angle beam scanning ability shall be designed.

In this paper, a new lens shaping method for thin and small lens curvature is proposed. Multi beam characteristics are compared by designing shaped lens antenna using the aperture distribution condition, Abbe’s sine condition and the newly proposed shaping method based on the straight-line concept. At first, lens shape design equations for three methods are shown. Three types of lens shapes are obtained by the Matrix Laboratory (MATLAB) program. Next, the multi beam radiation pattern is calculated by a commercial electromagnetic simulator FEKO.

## 2. Materials and Methods

### 2.1. Lens Antenna Structure and Parameter

**Figure 1:**
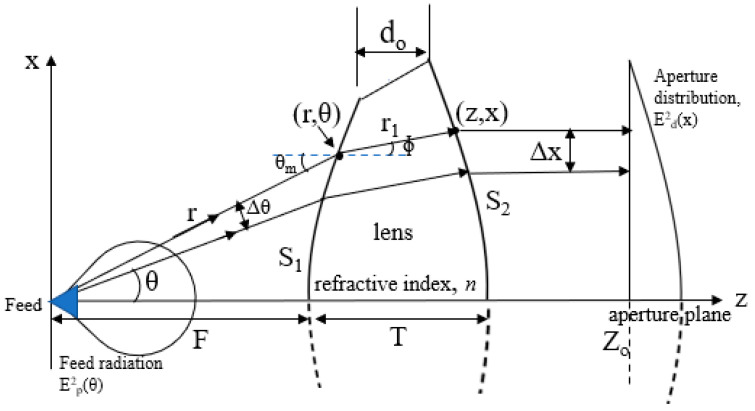
Design concept of lens shaping method.

The design concept and lens antenna parameters are shown in Figure 1. The ray tracing method based on Geometrical Optics (GO) is employed in the lens design. The lens is rotationally symmetrical around the z-axis, and the x-axis corresponds to the radial direction of the lens. The lens is composed of two surfaces, the inner surface (S1) and outer surface (S2). Radiated radio waves from the feed horn are expressed by rays diverging from the feed horn. Rays coming to the lens are refracted at the inner and outer surfaces. The ray emitted from the feed at an angle, θ, reaches S1 at the point indicated by (*r,θ*). After the ray is refracted at S1 by an angle ɸ, the ray reaches S2 at the point indicated by (*z,x*). All rays passing through the lens beam become parallel to the z-axis, which indicates that a flat wave front is formed. On the aperture plane, an illumination distribution, *E_d_^2^(x)*, is achieved depending on the feed horn radiation pattern, *E_p_^2^(θ)*. Refraction conditions on the lens surfaces and the aperture distribution condition are given by differential equations as shown in the next sub-section. By solving the simultaneous differential equations, shaped lens surfaces are obtained.

### 2.2. Lens Surface Design Equations

In order to clarify the proposed lens design method, conventional design methods such as aperture distribution condition and Abbe’s sine condition are explained. This section shows the equations used in designing the lens shape based on all three methods.

#### 2.2.1. Fundamental Ray Equations

There are three important expressions to represent the ray tracing principle, which are derived based on the Snell’s Law [24]. The Snell’s Law on the inner surface (S1) is given by Equation (1):

(1) $$\frac{dr}{d\theta }=\frac{nr\;\sin\left(\theta -ɸ\right)}{n\cos\left(\theta -ɸ\right)-1}.$$

The Snell’s Law on the outer surface (S2), is given by Equation (2), where *n* is the refractive index of the lens and ɸ is the refracted angle of the lens.

The expression for slope $\frac{dz}{dx}$ can be derived from the condition that all exit rays after refraction are parallel to the z-axis. The $\frac{dz}{dx}$ and $\frac{dx}{d\theta }$ shown in Equation (2) for variable change from *dx* to *dθ*.

(2) $$\frac{dz}{dx}=\frac{n\;\sin\;ɸ}{1-n\cos ɸ},\;\frac{dz}{d\theta }=\frac{n\;\sin\;ɸ}{1-n\cos ɸ}\frac{dx}{d\theta }$$

In the ray tracing calculation, the constant condition of the total electric path length, *L_t_*, is given by expression (3):

(3) $$L_{t}=r+\;nr_{1}+Z_{o}-Z,$$

(4) $$r_{1}=\frac{Z-r\cos\theta }{\cos ɸ},$$

where *Z_o_* is the aperture plane position and *Z* is the distance from inner surface at the edge to aperture plane, respectively. Equation (4) determines the ɸ value for a given *θ* value. Then, Equations (1) and (2) can be solved for the variable *θ*, if $\frac{dx}{d\theta }$ is known, which can be calculated from Equation (5).

#### 2.2.2. Aperture Distribution Condition

The following electric power conservation condition is given to obtain the differential $\frac{dx}{d\theta }$ equation.

(5) $$\frac{dx}{d\theta }=\frac{E_{p}^{2}\left(\theta \right)}{P}\frac{D}{E_{d}^{2}\left(x\right)}$$

Here, the total horn power, *P*, is given by Equation (6).

(6) $$P=\int E_{p}^{2}\left(\theta \right)d\theta$$

The total aperture power, *D*, is given by Equation (7).

(7) $$D=\int E_{d}^{2}\left(x\right)dx$$

**Figure 2:**
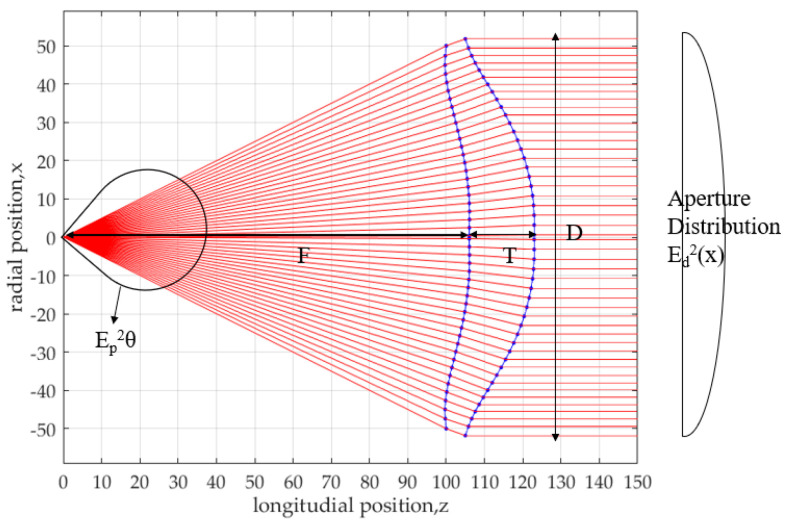
Lens shape based on the energy conservation law.

The fundamental parameters in lens shaping are the feed radiation, *E_p_*^2^(*θ*), and aperture distribution, *E_d_*^2^(*x*). The values of *E_p_*^2^(*θ*) and *E_d_*^2^(*x*) are given as follows:

(8) $$E_{p}^{2}\left(\theta \right)=\cos^{m}\left(\theta \right),$$

(9) $$E_{d}^{2}\left(x\right)={\left[1-\left(1-\frac{1}{C}\right)\left(\frac{x}{X_{m}}\right)\right]}^{p},$$

where *C* is the edge illumination of an aperture distribution. The value p determines the aperture distribution taper and *X_m_* is the maximum radius of the aperture. Differential Equations (1), (2) and (5) can be solved based on the constant path length condition of Equations (3) and (4). The performance of this method is determined based on the calculated or designed aperture distribution, *E_d_*^2^(*x*), for a given horn radiation pattern, *E_p_*^2^(*θ*). An example of a designed lens shape is shown in Figure 2. The newly developed MATLAB program will be discussed in Section 2.3, and the antenna parameters will be described in Section 2.4. The shapes of the inner and outer lens surfaces have special curvatures. During transmitting mode, all rays go into the aperture plane and become parallel to the horizontal axis. The spacing of rays is observed to be gradually increased towards the lens edge in order to achieve the aperture distribution taper.

**Figure 3:**
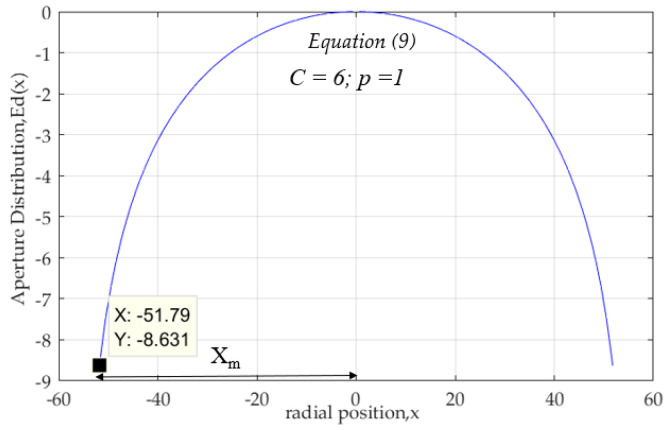
Aperture distribution on x-z plane.

The aperture distribution calculated by Equation (9) shows that the maximum radius of outer surface, *X_m_* = 51.79 mm, for tapered must be larger than 1 (C > 1). Here, C is the edge illumination (C = 6), and the edge level of the aperture electric field is −8.63 dB as shown in Figure 3.

#### 2.2.3. Abbe’s Sine Condition

Another method of obtaining the $\frac{dx}{d\theta }$ expression is through the Abbe’s sine condition as explained in [18], where the coma free condition is found for a limited scan. The condition, as expressed by Equation (10), is called Abbe’s sine condition.

(10) $$x=F_{s}\;\sin\;\theta$$

**Figure 4:**
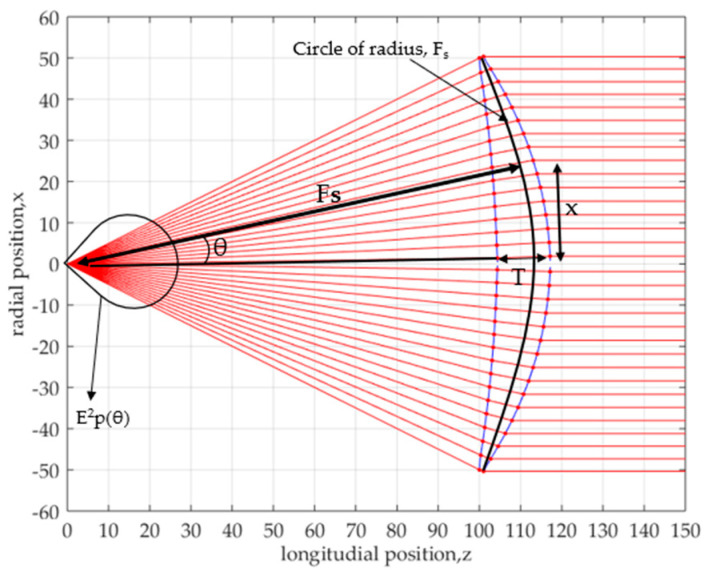
Abbe’s sine condition lens.

The meaning of this equation is shown in Figure 4. Based on this figure and by employing the Abbe’s sine condition, the crossing point of the incoming and the refracted rays should exist on the circle of radius, *Fs*. The differential form of Equation (10) if given by the next equation. The lens shape shown in Figure 4 is solved by Equations (1), (2) and (11), respectively.

(11) $$\frac{dx}{d\theta }=F_{s}\;\cos\;\theta$$

#### 2.2.4. Proposed Lens by Straight-Line Condition

As explained in Section 2.2.3, the differential equation $\frac{dx}{d\theta }$ of Abbe’s sine is derived from the condition that the refraction point is on the circle. As for the straight-line condition, small lens curvature and thin lens thickness are required; thus, the refraction point is expected to be better on the straight-line for a wide scanning beam.

**Figure 5:**
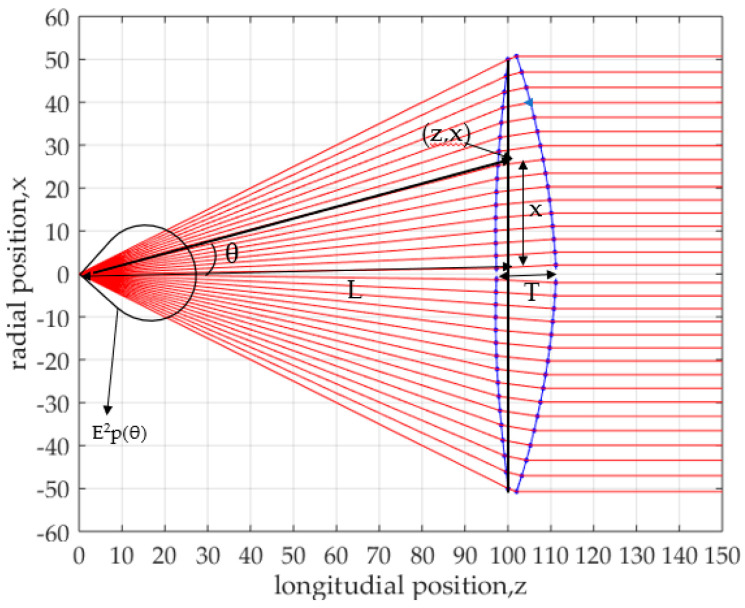
Straight-line condition lens.

By taking into account the radius of the circle in obtaining $\frac{dx}{d\theta }$ in the Abbe’s sine condition, the straight-line condition can also be obtained by the $\frac{dx}{d\theta }$ equation. The straight-line condition of the lens shape is shown in Figure 5, and the equation is given by Equation (12).

(12) x=  Ltanθ

The differential equation form is expressed by Equation (13), where *L* is the distance from the feed to the straight-line curve on the lens.

(13) $$\frac{dx}{d\theta }=L\;\sec^{2}\;\theta$$

From the developed MATLAB program, Equations (1), (2) and (13) have been solved, which has resulted in smaller lens thickness, T, and smaller curvature. Thus, this shape is suitable for a base station application.

### 2.3. MATLAB Program for Solving Differential Equations

**Figure 6:**
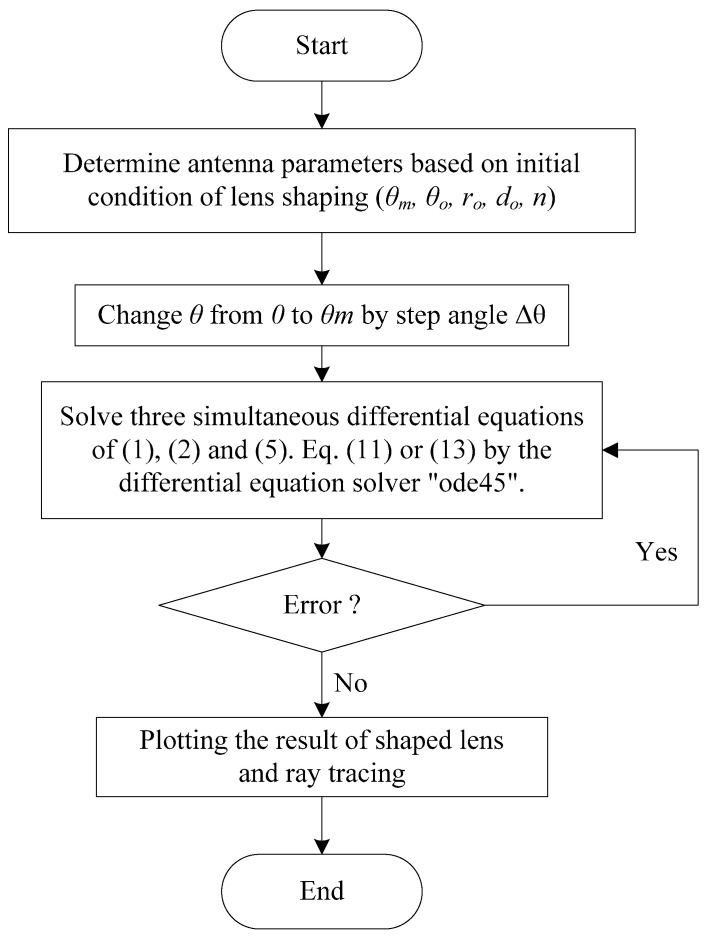
Flow chart of ray tracing program for lens antenna shaping.

**Figure 7:**
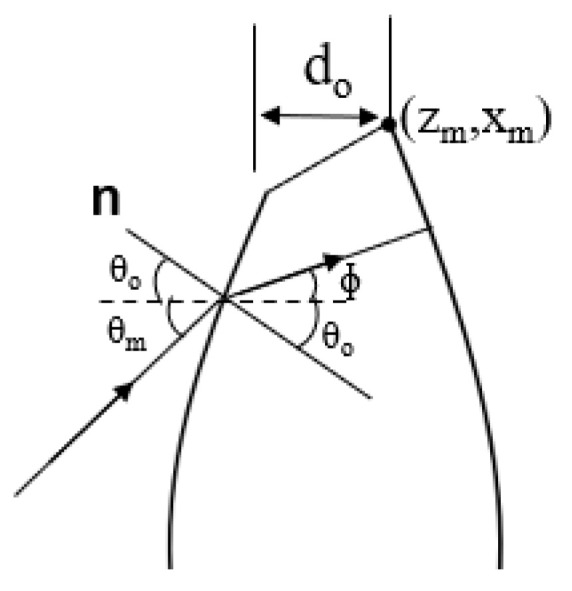
Initial condition of lens shaping.

In order to solve the three differential equations, a MATLAB program is developed. The flow of the developed program is explained by the flow chart shown in Figure 6. The parameter of the initial condition as shown in Figure 7 is determined. Here, *θ_o_* is an important parameter influencing the lens thickness. In accordance with the Δθ, changes of r and z are given by Equations (1) and (2), respectively. The change of *x* is determined by Equations (5), (11) and (13) for the design method of aperture distribution, Abbe’s sine condition and straight-line condition, respectively. As a result, the inner surface *(θ,r)* and the outer surface *(z,x)* are determined to design the lens surfaces and rays are plotted on the structure.

### 2.4. Structural Parameters for Lens Designing

In designing lens shapes shown in Figure 2, Figure 4 and Figure 5, the optimum antenna parameters used are shown in Table 1. As for common parameters, the lens diameter, D, is set to 100 mm and the feed radiation pattern is fixed. Other structural parameters of aperture distribution condition (ADC), Abbe’s sine condition (ASC) and straight-line condition (SLC) are independently determined based on the different design methods.

**Figure 8:**
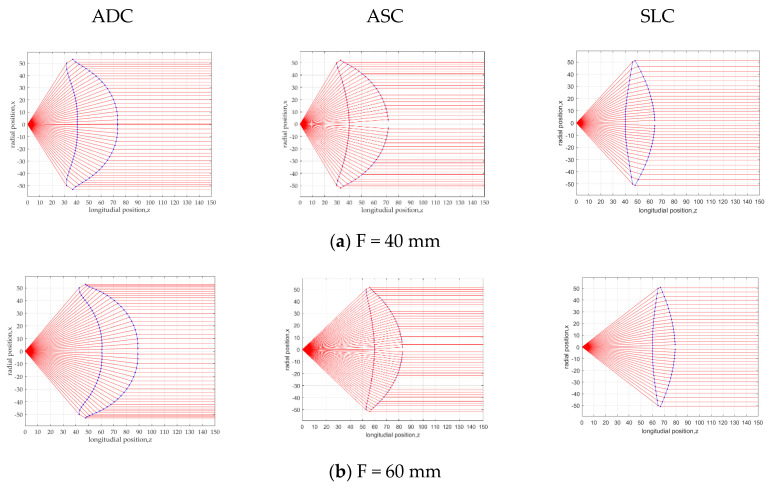
Lens structure for ADC, ASC and SLC at a focal length of (**a**) F = 40 mm, (**b**) F = 60 mm.

**Figure 9:**
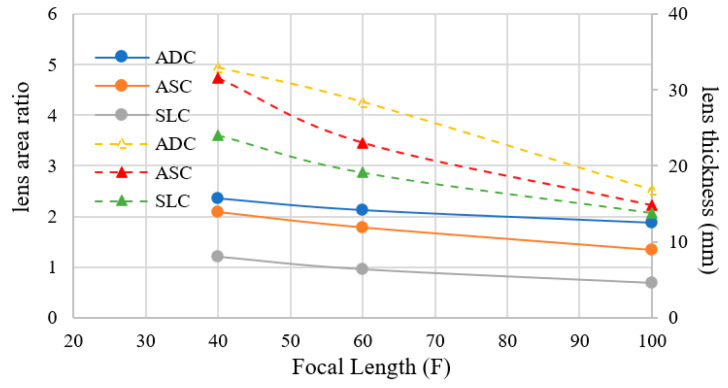
Lens area ratio and thickness at focal length F = 40 mm, F = 60 mm and F = 100 mm.

In order to clarify the feature of SLC, smaller focal length structures are shown in Figure 8. Calculation parameters are summarised in Table 2. From the figure, the lens thickness and area ratio of SLC become the smallest. In order to make clear the difference, the lens area and thickness are shown in Figure 9. It is clarified that SLC achieves the smallest lens area and thickness.

### 2.5. Radiation Pattern of a Feed Horn

**Figure 10:**
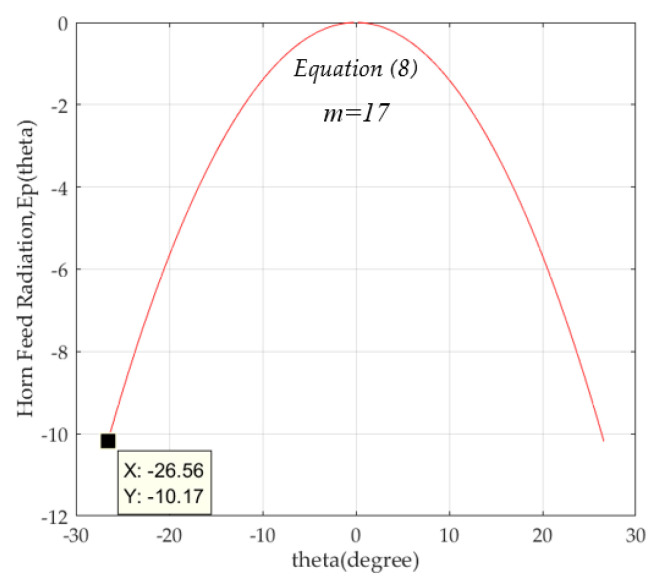
Feed horn radiation pattern, *E_p_*^2^(*θ*) = cos^17^θ, on x-z plane.

The feed horn radiation, *E_p_*^2^*(θ)*, employed in MATLAB is expressed by Equation (8) with radiation coefficient, m = 17, and the maximum angle from the feed horn is according to the lens shapes as tabulated in Table 1. The maximum power for lens illumination is about −10.17 dB, as shown in Figure 10. Based on the focal length to lens diameter ratio, F/D, the maximum angle from feed to lens edge is calculated to be about *θ_m_* = 26.56°.

### 2.6. Parameters of Electromagnetic Simulations

For validation, the shaped lenses developed in MATLAB are simulated by using an electromagnetic tool called FEKO to recognize its electrical performance. The numerical analysis in FEKO software is based on the Method of Moment (MoM) technique [25], and the antenna system consists of a horn antenna as the feeding element and a dielectric lens antenna. The use of the multilevel fast multipole method (MLFMM) becomes inevitable; thus, the surface equivalent principle (SEP) is employed for the feed horn and the dielectric lens body. The details of the simulation parameters are shown in Table 3. MoM is an accurate solver because it performs full wave analysis to derive rigorous solution for the complex model.

## 3. Simulation Results and Discussion

### 3.1. Feed Horn Design

**Figure 11:**
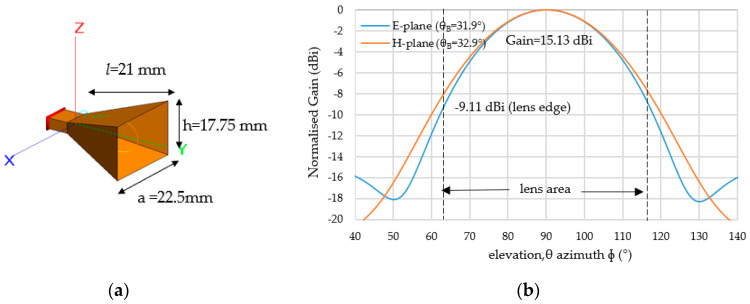
(**a**) Dimension of feed horn antenna and (**b**) radiation pattern (y-z plane).

In order to achieve the *E_p_*^2^(*θ*) radiation pattern as shown in Figure 10, a pyramidal horn antenna is employed in FEKO. The horn structure and size are shown in Figure 11a. The simulated radiation pattern for the E-plane and H-plane is shown in Figure 11b, which shows the obtained gain of 15.13 dBi with an edge level of −9.11 dB and −7.23 dB at the E-plane and H-plane, respectively.

**Figure 12:**
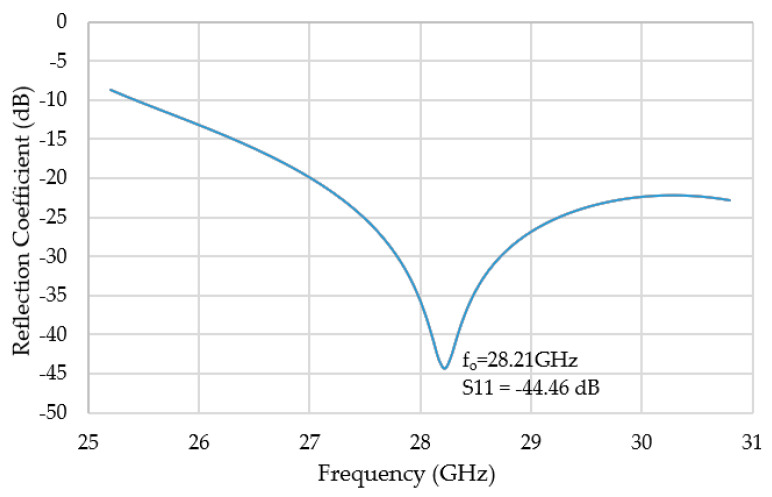
Reflection coefficient of a 28.21 GHz feed horn antenna.

Figure 12 shows that the reflection coefficient of a feed horn antenna is about −44.46 dB. The pyramidal feed horn is simulated from 25.2 GHz to 30.8 GHz, and the operating frequency, f_o_, is at 28.21 GHz.

### 3.2. Radiation Characteristics of the Centre Beam

**Figure 13:**
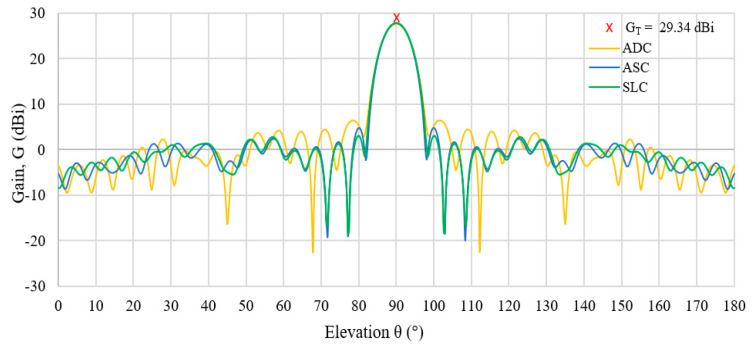
Radiation pattern for ADC, ASC and SLC lens with theoretical gain value, *G_T_* = 29.34 dBi.

The radiation patterns of all three lens antennas (ADC, ASC, SLC) are shown in Figure 13. All lenses have achieved the same main beam patterns and almost similar sidelobe levels. In order to examine the accuracy of the radiation patterns shown in Figure 10, the theoretical values of antenna gain and the beamwidth of uniform aperture distribution, as shown by Equations (14) and (15) [26], are calculated and compared. Here, *D* indicates the antenna diameter and *θ_BT_* is the half-power beamwidth (HPBW). The comparisons of the theoretical values and the simulation results are summarised in Table 4. Based on the data, the simulated beam widths are slightly increased for all lenses as compared to the theoretical beam width, *θ_BT_* = 6.25°, due to the tapered aperture distributions of the designed lens. The increased value of the taper aperture distribution has resulted in an increase of the beam width. In this paper, the taper aperture distribution, p, is 1; thus, the values of the beam width for all lenses are increased as compared to the beam width of uniform aperture distribution. In addition to that, the gain reduction from the theoretical value *G_T_* = 29.34 dBi is produced by the tapered aperture distributions when ADC, ASC and SLC gain are 27.56 dBi, 27.74 dBi and 27.69 dBi, respectively. Furthermore, the aperture efficiencies of 66.37% to 69.20% are also achieved.

(14) $$G_{T}={\left(\frac{\pi D}{\lambda }\right)}^{2}$$

(15) $$\theta_{BT}=58.4\frac{\lambda }{D}$$

### 3.3. Radiation Characteristics of Multi Beam

The multi beam antennas are capable of generating a number of synchronised and independent directive beams to cover the predefined angular and to provide a solution to overcome the shortcomings of the antenna with a single-directive beam. In this section, the radiation mode is explained for determining each of the feed coordinates at a specific angular range.

#### 3.3.1. Feed Positions for Multi Beams

**Figure 14:**
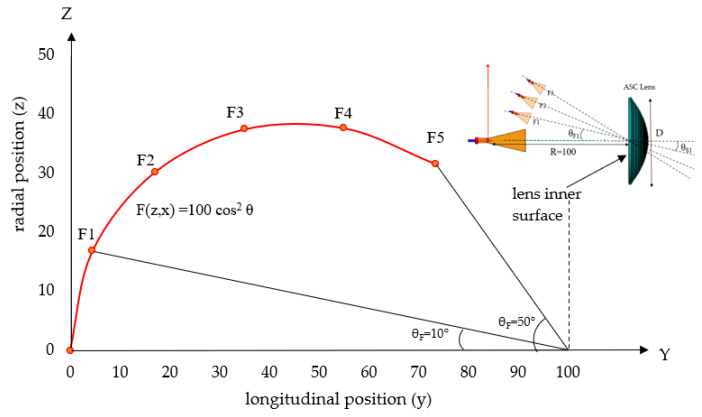
Locus of feed position.

The off-focus radiation patterns are investigated in the y-z plane. The relation of the feed positions with respect to the shift angles is shown in Figure 14. The feed positions of F4 and F5 seem to be approaching the inner surface of the lens. The locus of the feed position, *F(y,z)*, is determined below with *R* = 100 [27,28].

(16) $$F\;\left(y,z\right)=R\;\cos^{2}\theta_{F}$$

The feed coordinates are expressed by Equations (17) and (18).

(17) $$z=R\;\cos^{2}\theta_{F}\;\cos\theta_{F}+R$$

(18) $$x=R\;\cos^{2}\theta_{F}\sin\theta_{F}$$

Based on the equations, the feed coordinates are calculated and shown in Table 5 for *θ_F_* of the 10° step angle.

#### 3.3.2. Aperture Distribution Condition

**Figure 15:**
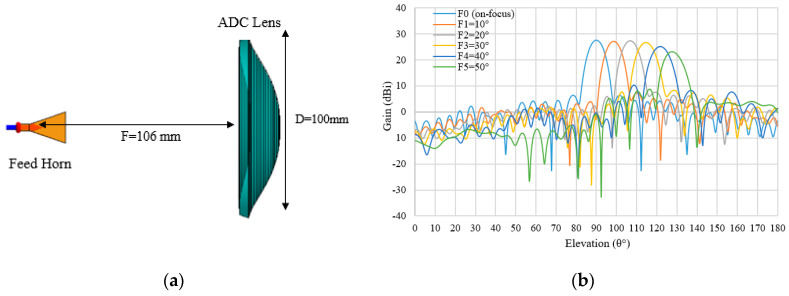
(**a**) ADC structure; (**b**) Multi beam radiation patterns for ADC lens shape.

The design parameters of the aperture distribution condition (ADC) are tabulated in Table 1, which is shown in Section 2.4. The ADC lens structure and multi beam radiation characteristic are shown in Figure 15a,b, respectively. The ADC lens produces a good multi beam pattern in the angle range of 40° (F1 to F4). It shows a slight drop in gain at the scanning angle *θ_F_* of 50° (F5). Moreover, the deterioration of the half power beam width is not significant with a difference of about 1.43° as compared to the on-focus feed (F0).

Table 6 summarizes the multi beam radiation characteristics and feed angles. The relation between the feed angle, *θ_F_*, and the beam shift angle, *θ_S_*, can be expressed as *θ_F_* = *aθ_S_*_._ The value of *a* is changed from 1.14 to 1.33 depending on the feed angles of 10° to 50°.

#### 3.3.3. Abbe’s Sine Condition

**Figure 16:**
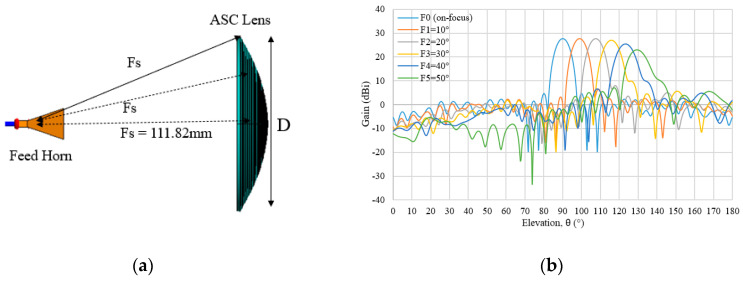
(**a**) ASC structure; (**b**) Multi beam radiation pattern for ASC lens.

In optics, Abbe’s sine condition is well known for designing a collimating lens for a limited scan. This ASC lens structure and multi beam radiation characteristic for on-focus (F0) and off-focus feeds (F1–F5) are shown in Figure 16a,b, respectively. A similar trend with the ADC lens has been discussed in Section 3.3.2, which shows a good radiation pattern produced by the ASC lens for angle range of 40°.

The multi beam characteristics of ASC are tabulated in Table 7, which specifies the shifted beam direction (*θ_S_*), HPBW(*θ_BS_*) and gain for each feed angle. Based on the table, a clear correlation between the feed angle, *θ_F_*, and shifted beam direction, *θ_S_*, is observed and it can be expressed as *θ_F_* = *bθ_S_*, where the *b* value is determined to be from 1.11 to 1.27. It can be clearly seen that there are no changes in gain for feed position, F1, but a slight decrement is observed from F2 to F4. Slight deterioration occurs in the main beam for F5 as the gain is dropped to 23.03 dBi from F0 with a gain difference of about −4.68 dBi. The maximum shifted beam direction is 39.3° when the feed angle is at 50°.

### 3.4. Proposed Lens by Straight-Line Condition

**Figure 17:**
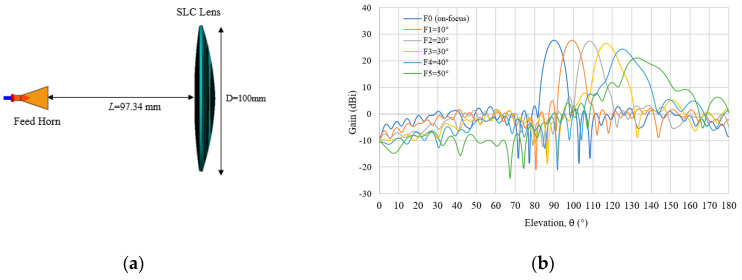
(**a**) SLC structure; (**b**) Multi beam radiation pattern for SLC lens.

The detailed lens parameters and structures calculated based on the newly proposed SLC method are tabulated in Table 1 and are shown in Figure 17a. This lens shape provides the lowest thickness as compared to the ADC and ASC lenses. The multi beam radiation characteristic for SLC is shown in Figure 17b, which is calculated for feed angle of *θ_F_* = 0° to 50°. From the figure, it is clearly seen that the tilt angle of 40° can also produce a good multi beam radiation pattern. At the beam direction of *θs* = 42.6°, the beam deformation becomes large.

The details of the SLC multi beam characteristics are shown in Table 8. It is found that F1 is similar to the ASC lens where there is no reduction in gain but it shows a slightly wider HPBW. It also has an almost similar relationship to the ADC and ASC, where the feed angle, *θ_F_*, and shifted beam direction, *θ_S_*, can be expressed as *θ_F_* = *cθ_S_*_._ The range value of *c* varies from 1.07 to 1.17. It can be seen that the feed angle, *θ_F_*, is larger than the shifted beam direction, *θ_S_*, for all lenses (ADC, ASC and SLC). This condition is demonstrated based on the principle of Snell’s Law when the waves are passing through between two boundaries from free space to a dielectric material. However, gain reduction becomes large for both feeds at F4 and F5, respectively.

**Figure 18:**
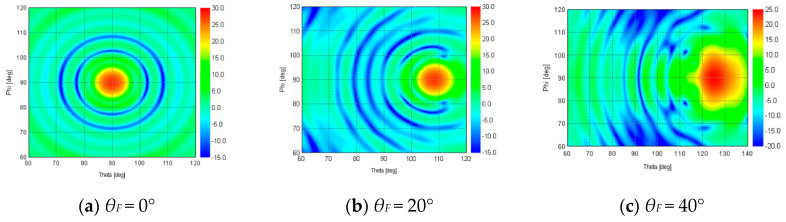
SLC 2D radiation pattern at feed angle (**a**) *θ_F_* = 0°, (**b**) *θ_F_* = 20°, (**c**) *θ_F_* = 40°.

In order to understand the beam shapes more in detail, 2D radiation patterns are shown in Figure 18. At a feed angle of *θ_F_* = 0° and 20°, the symmetrical main beam shapes in the theta (*θ*) and phi (ɸ) directions are achieved. In *θ_F_* = 40°, the main beam shape is not symmetrical. This beam shape deformation is caused by the aperture phase aberration in the off-focus feed.

**Figure 19:**
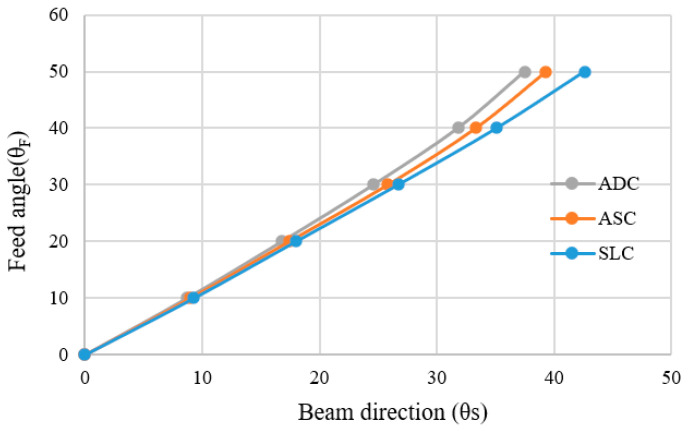
Feed angle and beam direction of ADC, ASC and SLC.

According to Table 6, Table 7 and Table 8, the beam directions of ADC, ASC and SLC from the feed angles 0° to 50° are shown in Table 9. The usefulness of the SLC design method is clarified, as maximum beam direction obtained is at *θs* = 42.6° as compared to ADC and ASC, where the beam directions are *θs* = 37.5° and *θs* = 39.3°, respectively. The correlation between the feed angle and the beam direction for all lenses is shown in Figure 19.

**Figure 20:**
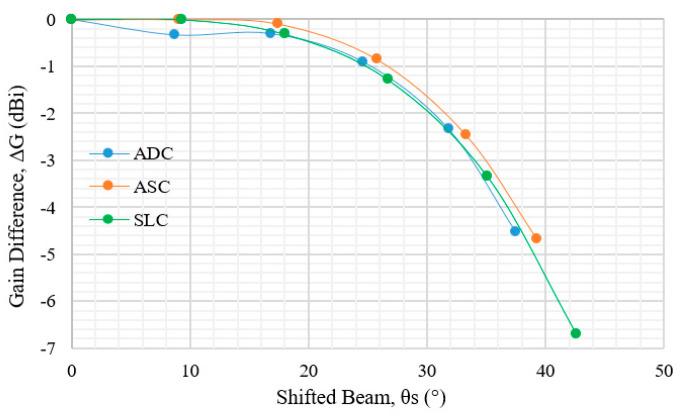
Gain difference of each shifted beam for ADC, ASC and SLC.

The correlation between the gain reduction and the shifted beam directions is shown in Figure 20. At beam directions of less than *θ_S_* = 40°, the gain reduction of ADC, ASC and SLC become similar. At *θ_S_* = 42.6°, the gain reduction increases to −6.69 dBi. From this figure, it is concluded that the proposed SLC achieves good multi beam radiation patterns as compared to the ADC and ASC lenses.

## 4. Conclusions

The new thin lens antenna has been designed by applying a straight-line condition. Multi beam radiation patterns are achieved and, as compared to the conventional lens antenna designed based on Abbe’s sine condition (ASC) and aperture distribution condition (ADC), this method also produced good results. For wide-angle beam scanning operation of up to 30° from the centre beam, good multi beam radiation patterns with small beam shape distortion are achieved. The usefulness of the newly developed shaped lens is ensured for multi beam radiation pattern characteristics in which an aperture efficiency of approximately 68.39% is achieved.

## Figures and Tables

**Table 1:** Dielectric lens antenna configuration.

Antenna Parameter	Dimension	Lens Shape	Dimension
**General Specification**		**Aperture Distribution (ADC)**	
Operating Frequency, f_o_	28.21 GHz	Focal Length, *F*	106 mm
Max. angle, *θ_m_*	26.56°	Length from horn to lens edge, *r_m_*	111.78 mm
Refractive index, n	2	Maximum radius of S2, *X_m_*	51.79 mm
Lens Diameter, D (10 λ)	100 mm	Total electrical length, *Lt*	266.98 mm
Initial condition of normal vector, *θ_o_*	10°	Lens Thickness, *T*	17.00 mm
Initial condition at lens edge, d_o_	5 mm	Aperture distribution, *E*^2^*_d_(x)*	Equation (9) and Figure 3
		**Abbe Sine Condition (ASC)**	
**Feed Horn {Ep^2^(θ)}**		Radius of circle, *Fs*	111.82 mm
Equation (8) and Figure 10		Maximum radius of S2, *X_m_*	50.13 mm
		Total electrical length, *Lt*	252.84 mm
		Lens Thickness, *T*	14.80 mm
		**Straight Line Condition (SLC)—Proposed**	
		Distance to lens, *L*	97.34 mm
		Maximum radius of S2, *X_m_*	50.36 mm
		Total electrical length, *Lt*	253.89 mm
		Lens Thickness, *T*	13.83 mm

**Table 2:** Lens antenna parameter for F = 40 mm and F = 60 mm.

Antenna Parameter	ADC		ASC		SLC	
	F = 40	F = 60	F = 40	F = 60	F = 40	F = 60
Max. angle, *θ_m_*	57.5	49.5	59.1	43.4	47.4	37.7
*m* of Equation (8)	5	10	5	10	5	10
*p* of Equation (9)	1	1	1	1	1	1

**Table 3:** Simulation parameters of horn feed and dielectric lens antenna.

Parameter	Specification
Computer	
Processor	Xeon 2.10 GHz
Random Access Memory	512 GB
Electromagnetic Software	FEKO 2019.2
Dielectric Lens Antenna	
Refractive index, n	2
Tan δ	0.0004
Mesh size	λ/8
Number of meshes	66,112
Feed Horn	
Material	PEC
Mesh size	λ/12
Number of meshes	18,532
Simulation Time (MoM)	3H

**Table 4:** Lens antenna performance for ADC, ASC and SLC.

Antenna Parameter	ADC	ASC	SLC
Gain of uniform aperture distribution, *G_T_* (dBi)		29.34	
Theoretical beam width, *θ_BT_*	6.25°		
Simulated Beam Width, *θ_BS_*	6.55°	6.40°	6.54°
Simulated Gain, *Gs* (dBi)	27.56	27.74	27.69
Gain difference	−1.78	−1.60	−1.65
Efficiency, *η*	66.37%	69.20%	68.39%

**Table 5:** Feed Coordinate.

Feed Angle (*θ_F_*)	Feed Coordinate	
	*y*	*z*
F1 = 10°	4.49	16.84
F2 = 20°	17.03	30.20
F3 = 30°	35.06	37.50
F4 = 40°	55.05	37.72
F5 = 50°	73.45	31.65

**Table 6:** Multi beam characteristics of ADC lens.

Feed Position	Feed Angle *θ_F_* (°)	Shifted Beam *θs* (°)	HPBW*θ_BS_*	Gain (dBi)	Gain Difference (dBi)
F0	0	0	6.54	27.55	0
F1	10	8.7	6.66	27.22	−0.33
F2	20	16.8	7.00	27.25	−0.30
F3	30	24.6	7.57	26.65	−0.90
F4	40	31.8	8.00	25.23	−2.32
F5	50	37.5	8.03	23.03	−4.52

**Table 7:** Multi beam characteristics of ASC lens.

Feed Position	Feed Angle *θ_F_* (°)	Shifted Beam *θs* (°)	HPBW*θ_BS_*	Gain (dBi)	Gain Difference (dBi)
F0	0	0	6.40	27.77	0
F1	10	9	6.50	27.77	0
F2	20	17.4	6.83	27.68	−0.09
F3	30	25.8	7.33	26.92	−0.85
F4	40	33.3	7.89	25.31	−2.46
F5	50	39.3	8.55	23.09	−4.68

**Table 8:** Multi beam characteristics of SLC lens.

Feed Position	Feed Angle *θ_F_* (°)	Shifted Beam *θs* (°)	HPBW*θ_BS_*	Gain (dBi)	Gain Difference (dBi)
F0	0	0	6.54	27.69	0
F1	10	9.3	6.63	27.69	0
F2	20	18	7.00	27.38	−0.31
F3	30	26.7	7.83	26.41	−1.28
F4	40	35.1	9.13	24.35	−3.34
F5	50	42.6	14.33	21.00	−6.69

**Table 9:** Beam direction of ADC, ASC and SLC lenses.

Feed Angle, *θ_F_*	Beam Direction, *θs*		
	ADC	ASC	SLC
0	0	0	0
10	8.7	9	9.3
20	16.8	17.4	18
30	24.6	25.8	26.7
40	31.8	33.3	35.1
50	37.5	39.3	42.6
